# Robust ultrasensitive tunneling-FET biosensor for point-of-care diagnostics

**DOI:** 10.1038/srep22554

**Published:** 2016-03-02

**Authors:** Anran Gao, Na Lu, Yuelin Wang, Tie Li

**Affiliations:** 1 Science and Technology on Micro-system Laboratory, Shanghai Institute of Microsystem and Information Technology, Chinese Academy of Sciences, Shanghai 200050, China

## Abstract

For point-of-care (POC) applications, robust, ultrasensitive, small, rapid, low-power, and low-cost sensors are highly desirable. Here, we present a novel biosensor based on a complementary metal oxide semiconductor (CMOS)-compatible silicon nanowire tunneling field-effect transistor (SiNW-TFET). They were fabricated “top-down” with a low-cost anisotropic self-stop etching technique. Notably, the SiNW-TFET device provided strong anti-interference capacity by applying the inherent ambipolarity via both pH and CYFRA21-1 sensing. This offered a more robust and portable general protocol. The specific label-free detection of CYFRA21-1 down to 0.5 fgml^−1^ or ~12.5 aM was achieved using a highly responsive SiNW-TFET device with a minimum sub-threshold slope (SS) of 37 mVdec^−1^. Furthermore, real-time measurements highlighted the ability to use clinically relevant samples such as serum. The developed high performance diagnostic system is expected to provide a generic platform for numerous POC applications.

Point-of-care (POC) diagnostics that offers great potential for early and rapid detection of diseases at lower costs in a portable format has emerged as an exciting field^1^,^2^,^3^. The development of POC diagnostics systems will provide the opportunities for better disease screening and monitoring at large scale^4^,^5^, whereas rapid, cheap, robust and accurate POC devices are in high demand. Silicon nanowire field-effect transistors (SiNW-FETs)^6^,^7^,^8^,^9^ are a highly attractive platform for POC testing because they are small, lightweight, low cost, and offer direct electrical readout, high sensitivity, and multiplexed detection^10^,^11^. By exploiting these attractive properties, SiNWs have been approved for a wide range of targets including DNA^12^, proteins^6^, small molecules^4^, and metal ions^13^. Silicon nanowire FETs fabricated with “top-down”^8^,^9^,^12^ techniques are particularly attractive due to CMOS-compatibility and mass manufacturing ability^8^,^14^. However, there are still some issues preventing use of conventional SiNW-FET biosensors in POC applications.

Specifically, conventional SiNW-FET biosensors face rising challenges in terms of device stability and poor disturbance resistibility^15^ when the device is scaled down. Thus, possible electrical cross talk and/or false-positive signals may occur and limit its utility in POC diagnostics. Zheng *et al*.^6^ have presented a possible solution for robust diagnosis of diseases using the incorporation of p- and n-type silicon nanowires. However, these SiNW devices were fabricated based on a “bottom-up” approach and rely on the assembly of grown SiNWs that inherently suffer from complex integration issues, which makes large scale production very difficult^16^. In addition, with a 60 mVdec^−1^ subthreshold slope (SS) limit at room temperature^17^,^18^, conventional SiNW-FET biosensors suffer from theoretical limitations on the maximum achievable sensitivity and minimum detection time^19^.

In this study, a SiNW-tunnel field-effect transistor (SiNW-TFET)-based biosensor employing band-to-band tunneling (BTBT)^20^,^21^ current injection mechanism was proposed. This idea has the potential to overcome these limitations while retaining all other advantages of conventional FET sensors^22^. TFET-based biosensor has been demonstrated earlier^19^. However, the literature only focused on the concept of TFET-based sensor, presented analytical and simulation-based studies. There is still little experimental results are available for TFET biosensor, which hinders its application in real world. The TFET is an inherent ambipolar device with p-type behavior with dominant hole conduction and n-type behavior with dominant electron conduction^17^,^23^. This favorable ambipolarity enables discrimination against false positives by correlating the response versus time from the two types of device behavior, and provides novel sensing strategies that result in a more robust device. For BTBT TFET, the tightest control is achieved in the quantum capacitance limit, which is most accessible to semiconductor nanowires^24^. Nanowires are preferred for TFETs as they provide better electrostatic gate control of the device channel due to the small wire diameter and provide improved electrostatics, and thus higher tunneling currents. Yet to date, there have been no systematic studies on silicon nanowire TFET based biosensor. The SiNW-TFET device can achieve SS < 60 mV dec^−1^ leading to substantially higher sensitivities versus conventional SiNW-TFETs. Therefore, the proposed SiNW-TFETs are potential candidates to replace conventional FETs for many applications.

## Results

### SiNW-TFET fabrication and characterization

In nanoelectronics, semiconducting nanowires can be fabricated by using either “bottom-up”^16^ or “top-down”^8^,^9^,^12^ methods. In this study, the SiNW-TFETs were “top-down” fabricated by applying a novel CMOS-compatible anisotropic wet etching approach. Rather than using expensive electron beam lithography, conventional optical lithography was combined with anisotropic wet etching via tetramethylammonium hydroxide (TMAH). The reproducible and well-controlled SiNW-TFET devices were manufactured in high yield by using the low-cost, CMOS-compatible fabrication approach (see Supplementary Fig. 1 for detailed fabrication process). Because TMAH etches Si (111) planes 100 times slower than other planes, this approach creates smooth edge imperfections not aligned to the (111) plane. The plot of undercut distance versus TMAH etching time shows the controllability of SiNW-TFET device fabrication (Supplementary Fig. 2). This approach overcomes limitations of traditional manufacturing methods^8^.

**Figure 1:**
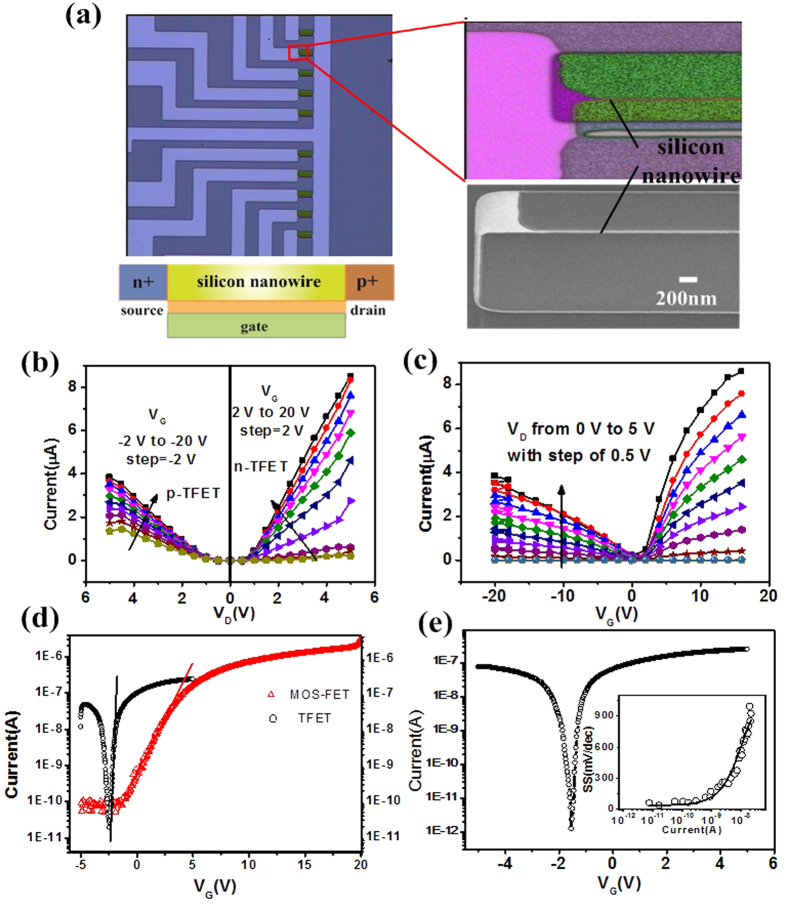
SiNW-TFET device fabrication and electrical characterization. (**a**) Optical micrograph of a fabricated SiNW array, zoomed-in optical micrograph, SEM image of a nanowire and schematic cross-section of a SiNW-TFET device. SiNW-based TFET is basically a p+−i-n+ structure (with n+ source, p+ drain, and intrinsic channel, for a p-type device) using B+ and P+ ion implantations to create heavily doped p+ and n+ regions. (**b**) The I_D_–V_D_ characteristics of a SiNW–TFET device for varying V_G_ (−2 V to −20 V with step = −2 V for p-channel TFET and 2 V to 20 V with step = 2 V for n-channel TFET). (**c**) I_D_–V_G_ characteristics of a SiNW-TFET device for varying V_D_ (0 to 5 V, step = 0.5 V). (**d**) I_D_–V_G_ curve of a SiNW-TFET device and a conventional n-type SiNW-FET device in log scale. Compared with conventional FET fabricated of the same batch which was fabricated with the same fabrication process and measured with the same configuration, the SS of SiNW-TFET was significantly decreased. (**e**) I_D_–V_G_ characteristic of a SiNW–TFETdevice for V_D_ = 1 V in log scale for a nanowire with a width of 70 nm, which provides a minimum SS of 37 mVdec^−1^ for the n-channel TFET at 300 K. The relation of the SS and current of n-channel TFET (inset) shows the SS of TFET is not a constant as in conventional FET but strongly depends on V_G_.

As indicated in Fig. 1a, the SiNWs are grouped in clusters (10 wires each). Such a layout is suitable for multiplexed detection because each cluster or wire may serve as one unit for sensing a particular molecule. The zoomed-in optical micrograph and scanning electron microscopy (SEM) image of a silicon nanowire marked with red rectangle box reveals that the surface of the SiNWs is fairly smooth and high quality. This is because of the planarization and slow anisotropic wet etching rate of Si (111) planes. (For a more detailed illustration of the structure, see Supplementary Fig. 3). The fabricated SiNWs are well ordered with triangular cross-section, small size, and high reproducibility (Supplementary Fig. 4). They also have dominant exposed Si (111) surfaces that are preferred for high efficiency surface functionalization^25^.

The back-gated SiNW-TFET with applied source (V_S_), gate (V_G_), and drain (V_D_) voltages is basically a p+−i−n+ structure (with n+ source, p+ drain, and intrinsic channel). In contrast to MOSFETs, in which charge carriers are thermally injected over a barrier, the primary injection mechanism of SiNW-TFETs is interband tunneling whereby charge carriers transfer from one energy band to another at a heavily doped p+−n+ junction. The tunneling current is proportional to the hole/electron transmission probability. This can be calculated with the Wentzel–Kramers–Brillouin (WKB) method (T_WKB_)^26^,^27^. The tunneling probability *T*_*WKB*_ can be calculated with the WKB approximation^28^ as 
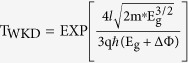
, where *m*∗ is the effective mass, *q* is elementary charge, *E*_*g*_ is the band gap and 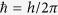 while *h* is Plank’s constant. The ΔΦ is the energy difference between the valence band in the channel and the conduction band in the source. *l* is the screening tunneling length, which describes the spatial extent of the transition region at the source-channel interface.

The silicon nanowire over a planar gate is electrostatically equivalent to a gate wrapped around the nanowire with oxide^29^. Considering the screening length in a one-dimensional wrap-around gate geometry, the screening length *l* reflects the device geometry under consideration, and it can be given by^26^,^30^: 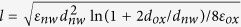, where *ε*_*nw*_ and *ε*_*nox*_ are the relative dielectric constants of the nanowire and the gate oxide, respectively. The *d*_*ox*_ is the gate oxide thickness, and *d*_*nw*_ is the nanowire diameter. In the case of a planar bulk TFET, the screening length *l* is given by^28^: 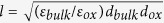, where 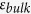 and 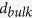 are the relative dielectric constant and thickness of the planar material. For a planar TFET there is a trade-off between realizing a high on-state current and a steep SS^28^. Due to an improved electrostatics, the one-dimensional TFET is possible to combine a high on-state performance with steep SS. Compared with planar TFET, the SiNWs are inherently small. An active channel yields very small *l* compared with planar TFET and *l* becomes even smaller as the SiNW scale decreases (Supplementary Fig. 5). Therefore, the BTBT probability *T*_*WKB*_ subsequently increases. Hence, the small nanowire size used here is favorable for enhancing the TFET device performance^31^,^32^,^33^.

The output characteristics of SiNW-TFET are shown in Fig. 1b in which the results demonstrate the high “on current”. The high “on current” demonstrates that the one-dimensional SiNW based TFET offers a high on-state performance with a steep SS^28^, which is superior to planar TFET. The transfer curves of the fabricated SiNW-TFET measured at room temperature are shown in Fig. 1c. As expected for TFETs, ambipolar characteristics with a strong dependence of I_D_ on V_G_ were observed. The tunneling current can flow at both negative and positive gate voltages, giving rise to ambipolar conduction, i.e. the device can be operated as an n- or p-channel TFET.

Compared with conventional SiNW-FET from the same batch, the SS of SiNW-TFET is clearly decreased (Fig. 1d). The SiNW-FET suffers from theoretical limitations on the minimum achievable SS (Supplementary). As shown in Fig. 1e, the I_D_–V_G_ characteristic for a nanowire with a width of 70 nm on the log scale provides a minimum SS of 37 mVdec^−1^ for the n-channel TFET at 300 K. This is much smaller than that reported for front-gated SiNW devices^10^. The p-channel TFET shows a minimum SS of 79 mVdec^−1^ indicating trap-assisted tunneling. The quality of the tunneling junction formed strongly and depends on the diffusion, activation, and segregation of dopants as well as defects remaining in the tunneling region^34^. Clearly the boron-implanted tunneling junctions are of significantly better quality than the phosphor-implanted junctions. The increase of SS with I_D_ is typical for TFETs (Fig. 1e, inset). In contrast to MOSFETs, the subthreshold swing of the TFETs is no longer a constant but depends strongly on V_G_. The smallest SS value occurs at the lowest device current. It is clear that the subthreshold swing increases quadratically with the gate voltage^35^. Therefore, it is highly desirable to use the average SS value rather than a point value to accurately evaluate the superior subthreshold characteristics of TFETs. The average SS is estimated to be 76 mVdec^−1^ (Supplementary). This is distinctly lower than for most reported SiNW-TFET devices^36^,^37^,^38^. Further improvements in fabrication conditions to form sharper tunneling junctions with more abrupt doping profiles may improve SS^34^,^39^.

### Sensing mechanism of the SiNW-TFET devices

**Figure 2:**
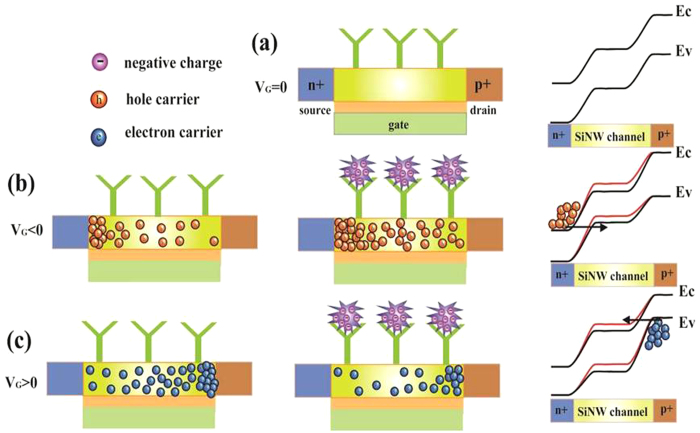
Schematic illustration of the working principle of SiNW-TFET for detection of electrical charged biomolecules. The SiNW-TFET device was functioned with specific receptors and passivated with ethanolamine for biosensing. The details can be found in the Experimental Section. (**a**) In the off-state, the tunneling barrier between the channel and n+/p+−doped source/drain regions is so large that tunneling is suppressed. (**b**) When V_G_ < 0 V, BTBT takes place at the channel/n+ source tunneling junction. The bonding of negatively charged biomolecules further decreases the BTBT barrier (red line). This increases the tunneling current of SiNW-TFET. (**c**) When V_G_ > 0 V, the tunneling junction is between the channel and the p+ drain region. This results in ambipolar conduction. The negatively charged biomolecule increases the BTBT barrier (red line) and reduces the tunneling current of the p-channel TFET.

To operate as a biosensor, the SiNW surface is first functionalized with specific receptors to capture the target biomolecules. The charged biomolecules being captured induce a gating effect and modulate the band-to-band tunneling barrier and hence the tunneling current. When V_G_ = 0, the TFET device is in the off state, and the tunneling barrier is so large that BTBT is suppressed (black line in Fig. 2a). This results in very small TFET off-state currents. In principle, the TFET is an ambipolar device, which means that conduction can occur in both negative gate voltage and positive gate voltage^40^. The p-type behavior occurs with dominant hole conduction, and n-type behavior occurs with dominant electron conduction. Applying a negative gate voltage increases the energy bands and reduces the tunneling barrier (black line in Fig. 2b). A conductive channel opens as soon as the channel valence band has been lifted above the source conduction band because carriers can now tunnel from the conduction band in the n+−doped source to the valence band in the channel. The charged biomolecules captured by receptors on SiNW-TFET further reduce the tunneling barrier (red line in Fig. 2b) and increase the BTBT current. If a positive gate voltage is applied, the energy band is decreased (black line in Fig. 2c) reducing the tunneling barrier width between the channel and p+−doped drain regions. Due to reduced energy barriers, the carriers can tunnel from the valence band in the drain to the conduction band in the channel, and the device is in the on state. The bonding of negatively charged biomolecule increases the band-to-band tunneling barrier (red line in Fig. 2c) reducing the tunneling current of the SiNW-TFET device.

### Ambipolar pH sensing

**Figure 3:**
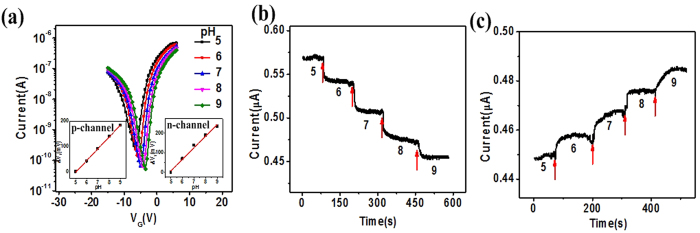
pH sensing with the SiNW-TFET device. (**a**) I_D_/V_G_ curve measured in solutions at different pH values (5, 6, 7, 8, 9) for V_D_ = 1 V. Plots of measured ΔV_th_ versus pH for n and p-channel device (inset). (**b,c**) Plots of SiNW-TFET current versus time for real-time pH sensing for V_D_ = 1 V and V_G_ = −1 V. (**b**) V_G_ = −3 V (**c**). Arrows mark the points when the solutions were changed. For the pH sensing measurement, 10 mM phosphate buffer solutions from pH 5 to 9 were used.

Unfunctionalized SiNW-TFET devices can be characterized as hydrogen ion sensors. Silicon nanowires with naturally occurring silanol (Si-OH) groups on its surface can be protonated and deprotonated by varying pH. Here, the surface charges change on the SiNW surface, which gate the TFET device to modulate the nanowire current^8^,^13^,^41^. The transfer curves for the SiNW-TFET under the different pH conditions are shown in Fig. 3a. The threshold voltage (V_th_) of the SiNW-TFET device was defined as the gate voltage at a drain current of I_D_ = 10^−9^ A based on the constant current method. As the pH value increases, the threshold voltage shifts to the positive direction. The pH sensitivity of 59 mVpH^−1^ for the n-channel device and 46 mVpH^−1^ for the p-channel device was obtained (Fig. 3a, insets). The SiNW-TFET device shows ambipolarity and conduction for both positive gate voltage and negative gate voltage^40^. As expected from the ambipolar characteristic, the SiNW-TFETs shows complementary signals from n- and p-channel TFET devices. This enables discrimination of electrical noise to facilitate object analysis (Supplementary Fig. 6).

The real-time responses of SiNW-TFET devices to five solutions with pH values varying from 5–9 are shown in Fig. 3b,c. A back gate bias was provided to control the initial operation regime of the SiNW-TFET based biosensor operating in p-channel or n-channel modes. The real-time current of silicon nanowires decreased stepwise with discrete pH changes from 5 to 9 when operated as an n-channel device. While the current of p-channel device increased with discrete changes from 5 to 9. At higher pH values, silanol groups are deprotonated. This makes the SiNW surface more negatively charged. For the n-channel TFET, the charges induce a negative surface potential increasing the band-to-band tunneling barrier and hence reducing the tunneling current (Fig. 3b), while the gate effect increases the current due to tunneling through the reduced barrier for the p-channel TFET (Fig. 3c).

### CYFRA21-1 biomarker detection

**Figure 4:**
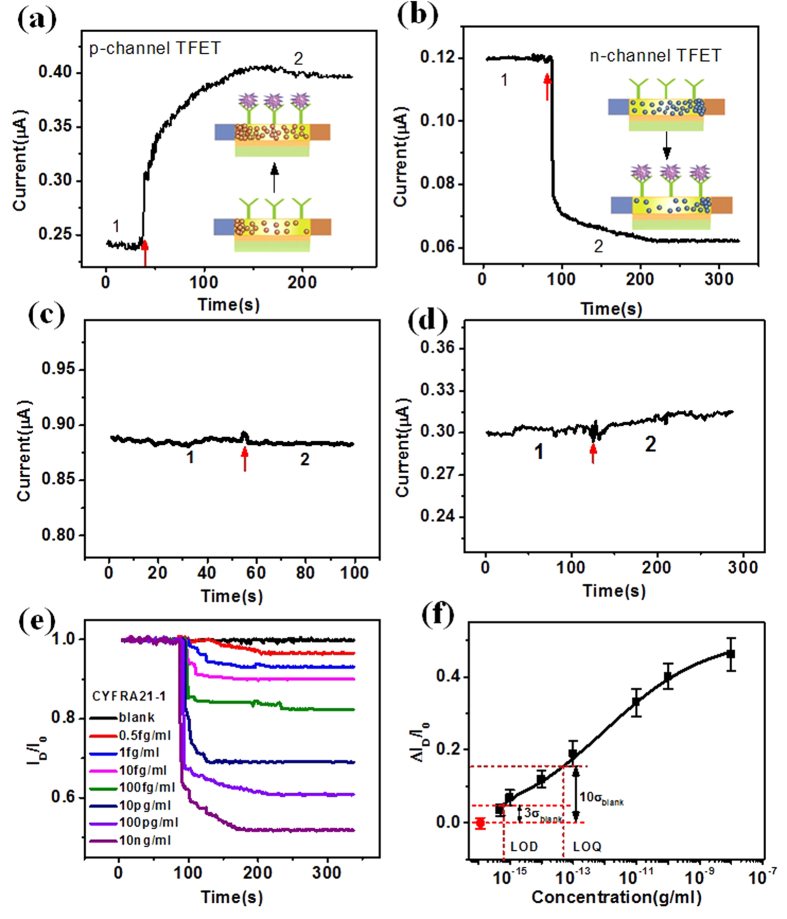
Real-time detection of CYFRA21-1 with the SiNW-TFET based biosensor. (**a,b**) Plots of SiNW-TFET current change versus time for real-time CYFRA21-1 detection for V_D_ = 1 V and V_G_ = −3 V(**a**) V_G_ = 2 V (**b**). Region 1 stands for the flow of buffer solution (0.01× PBS) and region 2 for the addition of 100 pgml^−1^ CYFRA21-1. The insets demonstrate the change of surface electrostatic environment and carrier in the SiNW channel. (**c,d**) Plots of current versus time for the unmodified SiNW-TFET (**c**) and the anti-CYFRA21-1-modified SiNW-TFET based sensor (**d**). Region 1 stands for the flow of buffer solution and region 2 for the introduction of 100 pgml^−1^ CYFRA21-1 (**c**), 1 nM of CEA (**d**). (**e**) Plots of normalized current change versus time with CYFRA21-1 at a series of concentrations (0.5 fgml^−1^, 1 fgml^−1^, 10 fgml^−1^, 100 fgml^−1^, 10 pgml^−1^, 100 pgml^−1^, and 10 ngml^−1^) for an anti-CYFRA21-1 modified SiNW device at V_D_ = 1 V and V_G_ = 2 V. (**f**) Plot of current change as a function of the logarithm of CYFRA21-1 concentration. The LOD and LOQ equal to three and ten times of the standard deviation of blank response.

In this study, we created a SiNW-TFET biosensor sensitive to CYFRA21-1 by immobilizing a specific antibody onto the SiNW surface. CYFRA21-1 is a biomarker of human lung cancer. It has proven to be the most sensitive biomarker in the diagnosis and prognostic of nonsmall cell lung cancer (NSCLC)^42^,^43^. Moreover, CYFRA21-1 may be helpful in identifying suspicious lung masses^44^. The detection of CYFRA21-1 can indicat the state of disease to greatly improve early diagnosis and prognosis of lung cancer. The antibody of CYFRA21-1 was attached on the SiNW surface according to the functionalization process. The contact angle measurements were made at different functionalized stages and fluorescence micrographs confirmed this high selective surface chemistry and successful attachment of CYFRA21-1 antibody (anti-CYFRA21-1) to the SiNW surface (Supplementary Table 1 and Fig. 7). We are performing an immunometric assay to measure fragments of cytokeratin 19 belonging to the acidic keratin^43^. CYFRA21-1 is negatively charged at our pH of 7.4 with an isoelectric point (pI) of 5.2^45^. Thus, it is expected that the current will increase upon binding of CYFRA21-1 to the antibody receptor on a p-channel SiNW-TFET resulting in increased tunneling through the reduced barrier (Fig. 4a); the opposite response was observed for a n-channel SiNW-TFET device (Fig. 4b). The n- and p-channel SiNW-TFET devices showed complementary currents indicating that both types of devices are highly responsive.

As a control, CYFRA21-1 at the same concentration (100 pgml^−1^) as in Fig. 4a,b flowed through an unmodified SiNW-FET without ethanolamine passivation. This led to negligible current change suggesting the absence of nonspecific binding of CYFRA21-1 to the SiNW surface (Fig. 4c). After a stable reading was achieved in the buffer solution without CYFRA21-1, the introduction of 10 ngml^−1^ CEA onto an anti-CYFRA21-1-functionalized SiNW-TFET surface produced a comparatively negligible change in current (Fig. 4d). This suggests that the nonspecific binding and adsorption of CEA protein molecules, even at higher concentrations, may be neglected. This demonstrates the high specificity of the SiNW-TFET based biosensor.

The sensitivity of the SiNW-TFET sensor was examined by challenging it with a series of concentrations of CYFRA21-1. The real-time response of nanowire current upon injection of various concentrations of CYFRA21-1 is illustrated in Fig. 4e. Known concentrations of CYFRA21-1in buffer were added after a stable reading using 0.01× PBS buffer as the diluent. The data were normalized by computing |I/I_0_| and plotted on the same axes for an effective comparison of the relative change in current of the SiNW-TFET device. The introduction of 0.5 fgml^−1^, 1 fgml^−1^, 10 fgml^−1^, 100 fgml^−1^, 10 pgml^−1^, 100 pgml^−1^, and 10 ngml^−1^ solutions of CYFRA21-1 resulted in a current change of ~3.5%, ~7%, ~10%, ~18%, ~31%, ~39%, and ~48%, respectively. The nanosensor could reliably detect CYFRA21-1 down to 0.5 fgml^−1^. The SiNW response as a function of the logarithm of CYFRA21-1 concentrations is illustrated in Fig. 4f. The electrical current change increased monotonously with the logarithm of CYFRA21-1 concentration. The direct, label-free detection of CYFRA21-1 down to 0.5 fgml^−1^ or ~12.5 aM demonstrates the ultrahigh sensitivity of this nanosensor.

The SiNW-TFET device reliability at femtogram per milliliter was further verified by repeated sensing experiment and by using p and n-channel TFET device through gate control (Supplementary Fig. 8). The complementary current changes demonstrated the reliability of SiNW-TFET device at ultra-low concentration. Limit of detection (LOD) and limit of quantification (LOQ) used to describe the smallest concentration of an analyte, are two important performance characteristics in method validation. The LOD and LOQ are calculated to be as 0.65 fgml^−1^ and 50 fgml^−1^ respectively, which were equal to three and ten times of the standard deviation of blank response (Fig. 4f). The CYFRA21-1 detection sensitivity is much higher than that of previously reported FET-type CYFRA21-1 sensor^46^.

With back gate control, the SiNW device configured as a p-channel TFET also revealed concentration-dependent current change and high sensitivity for molecular detection (Supplementary Fig. 9). The SiNW-TFET devices reveal complementary signals from n- and p-channel devices providing a simple and robust means of detection of false-positive signals from either electrical noise or nonspecific binding of protein onto nanowire surfaces. Furthermore, the sensitivity can also be enhanced with a higher percentage current change by correlating the response of n- and p-channel SiNW-TFET current change. By using the inherent ambipolar property of the TFET architecture, the detection is advantageous with strong anti-interference capacity to ensure stable operation under ambient noise and temperature variations, which results in a more robust and portable protocol.

**Figure 5:**
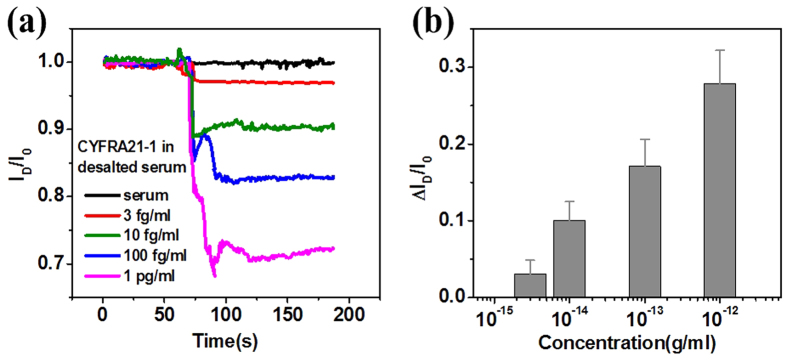
**CYFRA21-1 sensing in serum**. (**a**) Plots of normalized current change versus time with CYFRA21-1 in human serum at concentrations of 3 fgml^−1^, 10 fgml^−1^, 100 fgml^−1^, and 1 pgml^−1^ for the anti-CYFRA21-1-modified SiNW-TFET device at V_D_ = 1 V and V_G_ = 2 V. (**b**) Column plot of the relative current change as a function of the logarithm of CYFRA21-1 concentration.

It is important to accurately detect biomarkers in clinically relevant samples for biomedical POC applications. Hence, we challenged the SiNW-TFET sensor for the detection of CYFRA21-1 in human serum. The CYFRA21-1 was diluted to different concentrations of healthy human serum. An initial baseline for the current of the SiNW device was established by injecting a desalted serum solution not containing CYFRA21-1 (blank) onto the nanowire surface. Upon subsequent addition of the CYFRA21-1-containing serum solution, the current of the device immediately decreased and eventually stabilized. We found that this SiNW sensor could reliable detect as low as 3 fgml^−1^ CYFRA21-1 in serum samples (Fig. 5a). The concentration-dependent current change of the SiNW-TFET device in serum samples is shown in Fig. 5b. This clearly demonstrates its ability to detect biomarkers in real-sample assays. The results in real samples demonstrate that this is a simple and quick method for early detection and prognostic evaluation in lung cancer patients.

## Discussion

The SiNW-TFET biosensor was described for ultrasensitive and robust POC diagnostics. In contrast with other sensing technologies, the SiNW-TFET biosensor shows remarkable advantages. First, reproducible and well-controlled SiNW-TFETs can be manufactured in high yield via conventional silicon technology, allowing for large-scale and low-cost production. Second, the minimums SS of 37 mV/dec of SiNW-TFET device represents an improved operational conditions over conventional FET biosensors. LOD and LOQ are as low as 0.65 fgml^−1^ and 50 fgml^−1^ respectively, showing ultrahigh performance of the device. Third, the SiNW-TFET device can be operated as both n- and p-channel devices, which will result in opposing conductivity response change upon analyte binding. The two operation modes of device can be used to account for signal variation imposed by device fabrication. The developed SiNW-TEFT device with ultrasensitive, robust, real-time, small, versatile and low cost characters is beneficial for POC applications.

For the first time we have shown a label-free, ultrasensitive, electronic biosensor employing a BTBT-based SiNW-TFET fabricated with a CMOS-compatible anisotropic wet etching approach. The SiNW-TFET biosensors can not only provide substantial improvement in sensitivity but can also unambiguously distinguish noise from specific protein binding signals using favorable ambipolar effects of TFET for sensing. The SiNW-TFET devices reveal complementary signals from n- and p-channel devices. This provides a simple yet robust means of detection of false-positive signals to facilitate object analysis. Thus, we believe that the proposed ultrasensitive SiNW-TFET biosensors could pave the way for a paradigm shift in POC applications and hold great promise in simultaneous detection of multiple chemical and biological species.

## Methods

### Fabrication of SiNW-TFET device

A detailed fabrication flow for SiNW structure has been described previously^9^,^12^. The SiNW with length of 16 μm was fabricated with the fabrication flow. The BTBT current injection mechanism of SiNW-TFET device was realized by separate ion implantation for source and drain (phosphor doping for source and boron doping for drain and intrinsic SiNW channel). In order to decrease the damage to top silicon layer and obtain the uniform ion distribution, semiconductor simulation software Silvaco was used to optimize the parameters of doping and annealing. Specifically, a phosphor adulterant with doping dose 5 × 10^15^ cm^−3^, energy 40 keV at the source and a boron doping of 6 × 10^15 ^cm^−3^, energy 70 keV for drain were performed separately. After annealing at 1100 °C nitrogen for 30 min, an effective contact region with a concentration of about 10^20 ^cm^−3^ was formed for the p-i-n TFET device. The Cr/Au layer was finally deposited on top of silicon contact region by using magnetron sputtering and lift-off technique, making reliable ohmic contact between Au and Si (Supplementary Fig. 10).

### SiNW surface modification

Surface modification of the SiNW-TFET was performed based on the protocol described in our previous work^9^. The 3-aminopropyltriethoxysilane (APTES) was used to convert the surface silanol groups to amines followed by reaction with the biofunctional linkerglutaraldehyde. Then the CYFRA21-1antibody was attached on the SiNW surface, and the unreacted aldehyde groups were passivated with ethanolamine. The freshly prepared SiNW-TFET devices proceeded immediately to the electrical sensing test.

### General sensing apparatus and parameters

Electrical measurements for all sensing experiments were carried out by using a Keithley 4200 semiconductor parameter analyzer (Keithley Instruments Inc., Cleveland, OH). For CYFRA21-1 sensing, we used V_D_ = 1V, V_G_ = 2V for SiNW-TFET to work as an n-channel device and V_D_ = 1 V and V_G_ = −3 V for the p-channel device, respectively. Solutions of CYFRA21-1 were prepared by dissolving CYFRA21-1 into 0.01× PBS and serially diluting to various concentrations. The buffer solution (0.01× PBS) with volume of 5 μl was first introduced on SiNW surface to establish an initial baseline for SiNW-TFET device. Then 5 μl solution containing CYFRA21-1 was injected onto the nanowire. The current through the SiNW-TFET device was measured relative to the baseline current for different concentrations of CYFRA21-1.

### Serum assays

In serum assays, CYFRA21-1 was dissolved in 100% human serum and serially diluted to different concentrations. The same protocol was used as in the buffer solution for the measurements.

## Additional Information

**How to cite this article**: Gao, A. *et al*. Robust ultrasensitive tunneling-FET biosensor for point-of-care diagnostics. *Sci. Rep.* **6**, 22554; doi: 10.1038/srep22554 (2016).

## Supplementary Material

Supplementary Information
