# Supplementary material for: Robust ultrasensitive tunneling-FET biosensor for point-of-care diagnostics
Source: Sci Rep. 2016 Mar 2;6:22554. doi: 10.1038/srep22554 (PMC4773835; doi:10.1038/srep22554)
Supplement: Supplementary Information [file srep22554-s1.pdf]

Supplementary Information

Robust ultrasensitive tunneling-FET biosensor for point-of-care diagnostics

Anran Gao, Na Lu, Yuelin Wang*, Tie Li*

Science and Technology on Micro-system Laboratory, Shanghai Institute of Microsystem and Information Technology, Chinese Academy of Sciences, Shanghai 200050, China

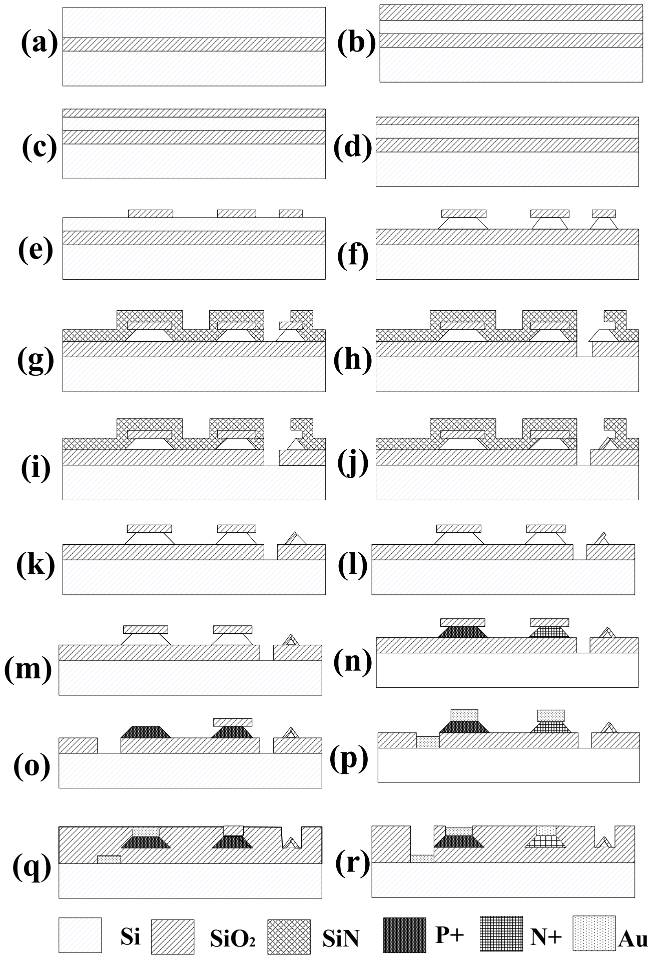

Figure 1. The fabrication process of SiNW-TFET device.

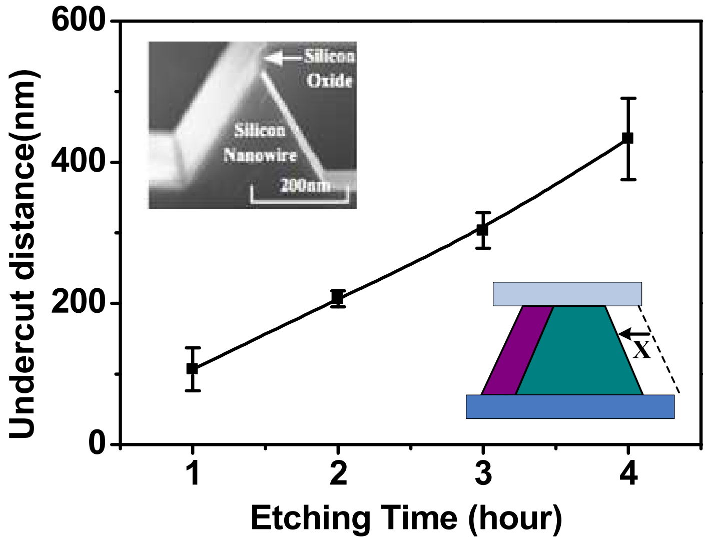

Figure 2. Polt of the undercut distance versus the etching time.

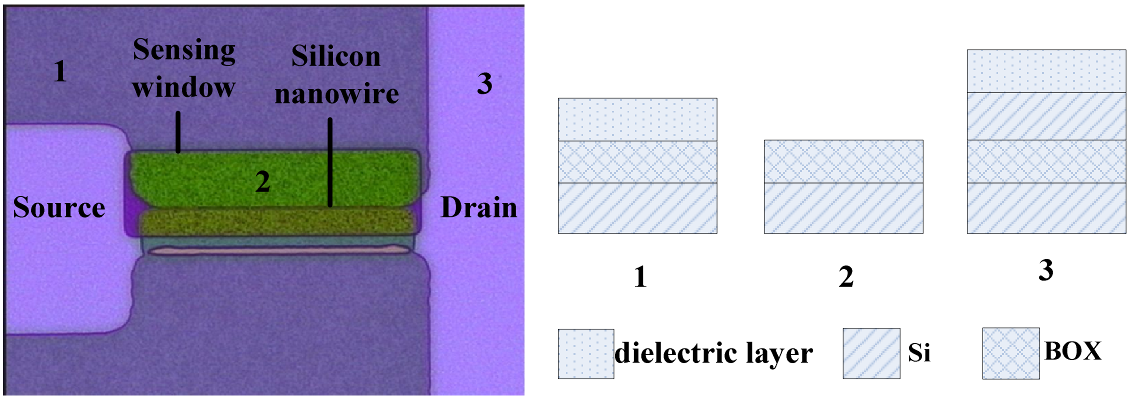

Figure 3. Schematic illustration of the fabricated SiNW-TFET device and structure.

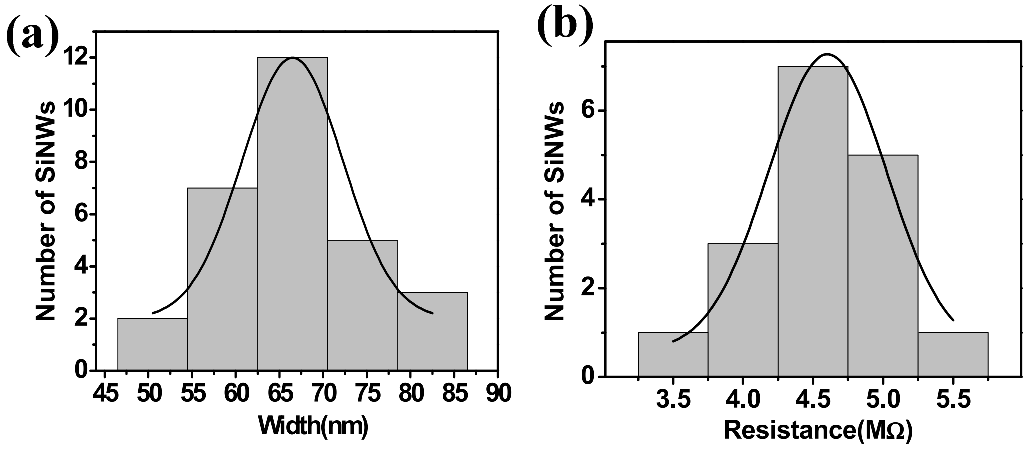

Figure 4. The reproducibility of the fabricated SiNWs. (a) Distribution of the width of a batch of SiNWs

was shown in columns. (b) Column plot of the conductance of fabricated SiNWs.

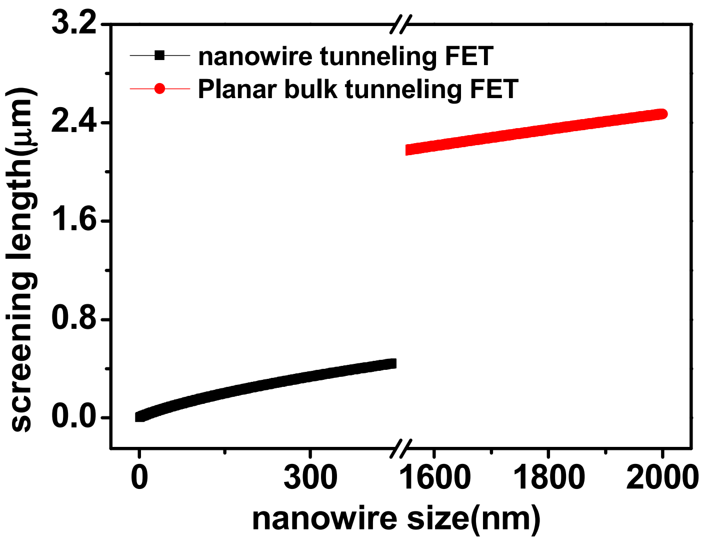

Figure 5. The screening tunneling length comparison of 1D nanowire tunneling FET and planar bulk tunneling FET.

Subthreshold slope (SS) of MOSFET

For metal–oxide–semiconductor FET (MOSFET), SS is driven by:

$$SS = \frac{dV_G}{d(\lg I_D)} \cong \left(1 + \frac{C_d}{C_{ox}}\right) \ln 10 \frac{kT}{q} \qquad (1)$$

where V_G is the gate voltage, I_D is the drain current, k is Boltzmann constant, T is temperature, q is the elementary charge of an electron, kT/q representing the thermal voltage, and C_d and C_{ox} are the depletion and the oxide capacitances, respectively. $\ln 10 \frac{kT}{q} \cong 60mV/dec$ at room temperature. Therefore, the current-switching process involves the thermionic (temperature-dependent) injection of electrons[1] over an energy barrier, setting a fundamental limit to the steepness of the transition slope from the off to the on state[2].

Numeric Calculation of average SS

Unlike in the case of MOSFETs, the subthreshold swing of TFETs is not constant, but rather is

highly dependent on the gate voltage. The subthreshold swing increases quadratically with the gate voltage[3]. It is therefore highly desirable to use the average value of the subthreshold swing, rather than a point value, to accurately evaluate the subthreshold characteristics of TFETs. The average subthreshold swing is then defined as[4]:

$$S_{ave} = \frac{|V_T - V_{\min}|}{\log\left(I_{D-V_T}\right) - \log\left(I_{\min}\right)} \tag{2}$$

where V_T is the threshold voltage, V_{min} and I_{min} correspond to the valley voltage and valley current of the transfer curves, respectively.

Since there is no standard extraction method for the threshold voltage of the TFET, the gate voltage at a drain current of $I_D = 10^{-9}$ A is taken to be the threshold voltage based on the constant current method. The normal n-channel mode behavior with a threshold voltage of −1.3 V is clearly shown in Fig. 1e. Therefore, the average subthreshold swing is estimated to be 76 mVdec^{-1}

pH sensing at different gate bias

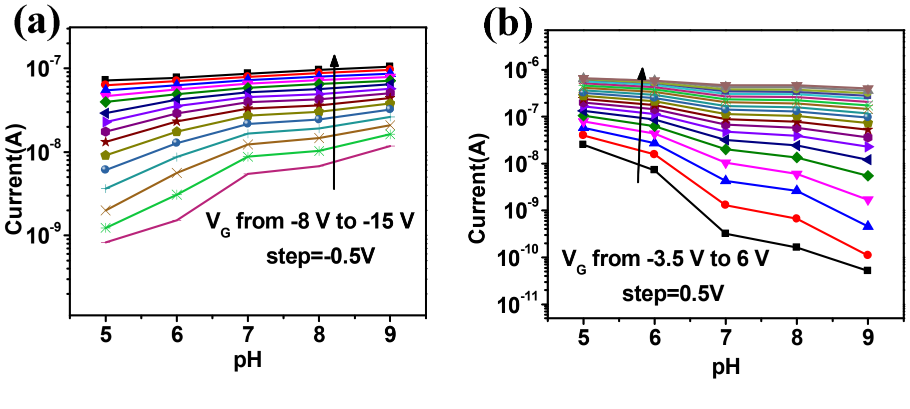

Figure 6. Current of SiNW-TFET nanosensor as a function of pH value at different gate bias for p-channel TFET (a) and n-channel TFET (b).

SiNW surface modification

Table 1. The measured contact angles for silicon surface at various functionalized stages.

**surface**	**contact angle (deg)**
soxide surface	101
hydroxyl-terminated surface	0
amine-terminated surface	53
well-ordered amine-terminated surface	71
aldehyde group terminated surface	61
antibody of CYFRA21-1 modified surface	70

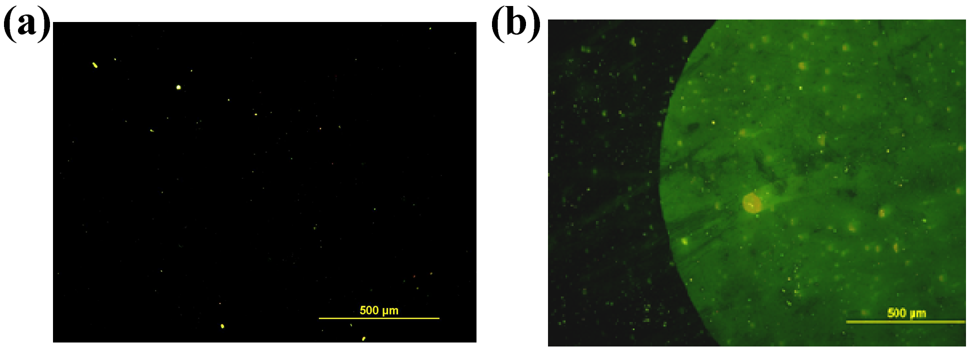

Figure 7. Fluorescence image of unmodified blank silicon surface (a) and the surface with modification (b), after introduction of solution containing antibody of CYFRA21-1 on the surface for 2 hours followed by thoroughly wash with clean water.

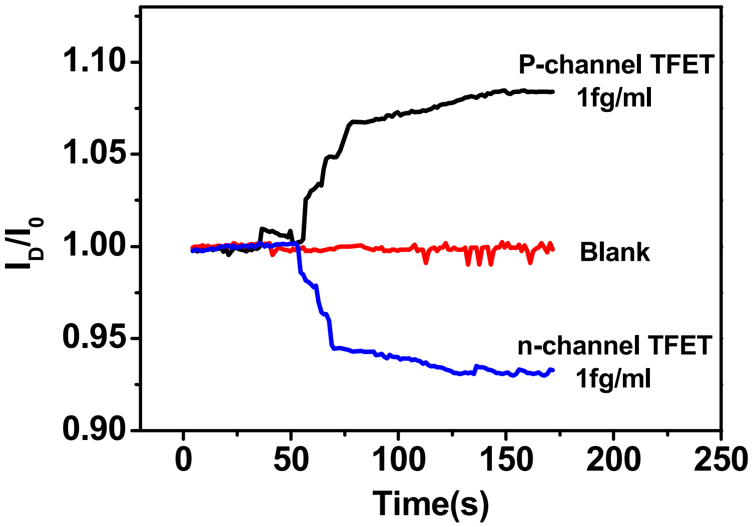

Figure 8. The reliability of SiNW-TFET device for CYFRA21-1 detetction at femtogram per milliliter.

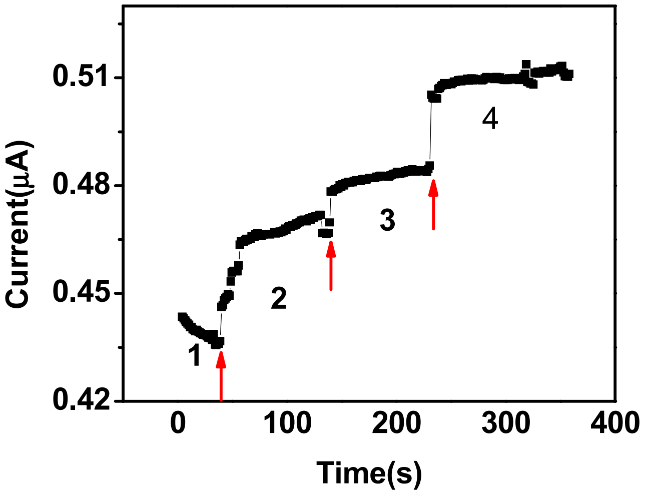

Figure 9. Plots of SiNW-TFET current change versus time for real-time CYFRA21-1 detection for V_D=1 V and V_G=-3 V, where region 1 stands for the flow of buffer solution, region 2 for the addition of 1fg/ml CYFRA21-1, region 3 for the addition of 10fg/ml CYFRA21-1 and region 4 for the addition of 100fg/ml CYFRA21-1.

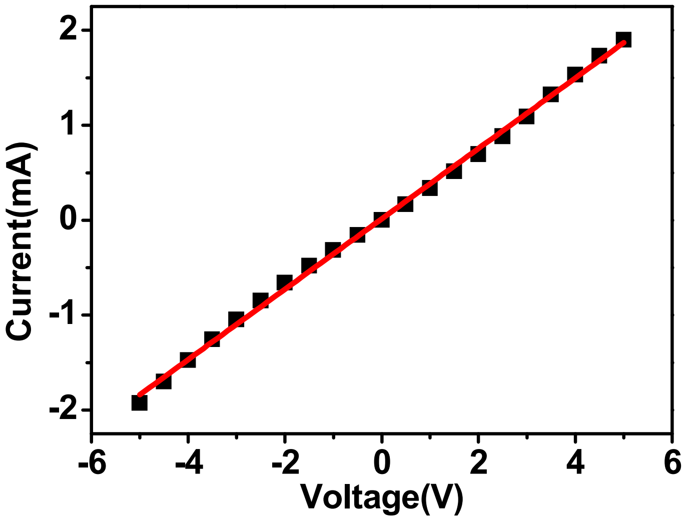

Figure 10. The relationship between applied voltage and current for doped silicon, showing ohmic contact between doped source/drain and metal electrodes.

References

1. Sze, S.M., Crowell, C.R., Carey, G.P. & Labate, E.E. Hot-Electron Transport in Semiconductor-Metal-Semiconductor Structures. *J Appl Phys* **37**, 2690 (1966).

2. Ionescu, A.M. & Riel, H. Tunnel field-effect transistors as energy-efficient electronic switches. *Nature* **479**, 329-337 (2011).

3. Boucart, K. & Ionescu, A.M. Double-gate tunnel FET with high-kappa gate dielectric. *Ieee T Electron Dev* **54**, 1725-1733 (2007).

4. Lee, M. et al. Silicon nanowire-based tunneling field-effect transistors on flexible plastic substrates. *Nanotechnology* **20,** 455201 (2009).
